# Associations between Canadian deprivation indices and acute stroke outcomes post endovascular thrombectomy - A retrospective cohort study

**DOI:** 10.1177/15910199251396174

**Published:** 2025-12-02

**Authors:** Matthew C So, Nishita Singh, Johanna M Ospel, A Zohaib Siddiqi, Nada Dahroug, Santhosh Annayappa, Kamran Zahid, Susan Alcock, Roman Marin, Ankur Wadhwa, Claudia Candale-Radu, Anurag Trivedi, Esseddeeg Ghrooda, Naveed Akhtar, Mohammed Suheel, Joseph Silvaggio, Jai Shankar, Nima Kashani

**Affiliations:** 1 Department of Radiology, 423134 University of Manitoba, Winnipeg, Canada; 2 Department of Neurology, Rady Faculty of Health Sciences, 12359 University of Manitoba, Winnipeg, Canada; 3 Department of Neurosciences, 30262 University of Calgary, Calgary, Canada; 4 Department of Medical Imaging, University of Toronto, Toronto, Canada; 5 Max Rady College of Medicine, 12359 University of Manitoba, Winnipeg, Canada; 6 Department of Neurosurgery, 12359 University of Manitoba, Winnipeg, Canada

**Keywords:** Endovascular thrombectomy, mechanical thrombectomy, acute ischemic stroke, socioeconomic status, deprivation index

## Abstract

**Background:**

Socioeconomic status (SES) has long been recognized as an important determinant of ischemic stroke outcomes, with increased stroke severity and mortality found in patients with lower SES. However, the impact of SES on outcomes post-endovascular thrombectomy in Canada remains largely unknown.

**Methods:**

This study is a retrospective cohort analysis of patients from 2015–2024 who received endovascular thrombectomy for large vessel occlusion in Manitoba, Canada (study catchment area 650,000 km^2^, population 1.4 million). Patient residence postal codes were matched with neighborhood-level socioeconomic factors using the Canadian Material and Social Deprivation Index and the Canadian Index of Multiple Deprivation (CIMD). Regression analyses adjusting for baseline demographics, geographic variables, presenting National Institutes of Health Stroke Scale (NIHSS), and time to angiography were conducted to analyze the association between socioeconomic factors and a binarized 90-day modified Rankin Scale (mRS).

**Results:**

Overall, 793 patients (51% females, median age 74 years) were included. Unadjusted analyses showed a positive association between poor clinical outcomes and increased economic dependency scores in the CIMD index (OR = 1.18 [95%CI 1.03, 1.37]), as well as decreased CIMD ethno-cultural composition scores (OR = 0.84 [0.73–0.98]). Adjusted analysis did not show consistent associations between procedural outcomes or functionally independent outcomes at 90 days.

**Interpretation:**

This Canadian provincial stroke registry study showed SES-related discrepancies in stroke outcomes on an unadjusted basis, but no definite discrepancies after adjustment for age, presenting severity, and time to angiography. These findings suggest that SES-related differences in these variables mediate the observed relationship between SES and poor clinical outcomes.

## Background

Acute ischemic stroke (AIS) is the third leading cause of death in Canada and responsible as one of the largest contributors to disability, reduced quality of life, and financial burden to the healthcare system.^1^ While global data suggests that the incidence and mortality rate of AIS has been decreasing over time, Canadian data suggests an increase in the crude population suffering from AIS, likely due to an aging and growing population.^2–4^ Global data additionally suggests a greater reduction in health-related quality of life measures in low socioeconomic status (SES) patients compared to high SES patients.^5^ Inadequate control of cardiovascular risk factors in low SES groups may explain poorer AIS outcomes in low SES groups within high-income countries such as Canada.^6^

In the past, the prognosis of AIS was dismal, particularly when caused by a large vessel occlusion. However, since 2015, endovascular thrombectomy (EVT), with or without intravenous thrombolysis, has become the standard of care for eligible patients with large-vessel occlusion (LVO),^7,8^ which has dramatically improved post-stroke outcomes. Nevertheless, still, 54% of LVO patients do not achieve a good outcome despite modern endovascular and pharmacological therapies.^7^ Furthermore, access to EVT can be heavily influenced by socioeconomic status (SES), a well-recognized determinant of population health.^9^ Individuals with lower SES not only face a higher risk of experiencing a stroke, but also present with more severe neurological deficits as reflected by higher National Institutes of Health Stroke Scale (NIHSS) scores at admission, and experience poorer clinical outcomes.^10^ These stroke outcomes mirror those in myocardial infarction, with lower SES associated with higher short-term mortality as well as lower cardiac catheterization and revascularization rates.^11^ Similarly, increased prevalence of major trauma is noted in socioeconomically deprived areas.^12^

In Canada, studies from Ontario and Alberta reveal socioeconomic disparities in stroke care, with patients from lower SES groups being less likely to receive thrombolysis or EVT.^13^ These findings highlight the importance of exploring how SES influences stroke outcomes post endovascular treatment. In the current study, we examine the influence of SES on health care outcomes in patients undergoing EVT at a provincial comprehensive stroke center in Manitoba, Canada, a province with vast geographic and economic disparities.^14,15^ Using provincial-level data from 2015–2024, this study explored insights into whether SES impacted post-stroke outcomes across the province of Manitoba.

## Methods

This study investigates the relationship between SES, encompassing material and social deprivation factors, as analyzed using the Material and Social Deprivation Framework (MSDI) framework provided by Blaser et al.,^16^ as well as the Canadian Index of Multiple Deprivation (CIMD)^16,17^ and neighborhood before-tax income, and functional post stroke health outcomes as measured by the 90-day modified Rankin Scale (mRS), in Manitoba's AIS patients which underwent thrombectomy.

### Stroke data sourcing

This retrospective cohort study includes data from the Optimizing Patient Treatment in Major Ischemic Stroke With EVT (OPTIMISE) registry collected between January 2015 and May 2024 of all patients that underwent EVT at the Health Sciences Centre (HSC) in Winnipeg, Canada. Ethics was approved under the OPTIMISE registry (HS22427 [H2018:506]). Health Sciences Center is the sole tertiary care hospital with the only biplane angiography suite within the province of Manitoba and serves the entirety of the province and neighbouring parts of Ontario and Nunavut. Data from 838 consecutive patients with ischemic stroke that underwent EVT were gathered. The exclusion criteria included: no fixed home address or home address outside of Manitoba, Canada and missing mRS scores. Each patient's primary residential postal code was matched to longitude and latitude coordinates using data from the Canadian Postal Code Conversion File, and subsequently straight-line distances between the location of origin and the emergency department of Health Sciences Center (the main EVT center) in Winnipeg were computed. Functionally independent “good” outcomes were defined as mRS 0–2, and a “poor” outcome was defined as mRS 3–6.

### Deprivation indices

This analysis used neighborhood-level indices of socioeconomic status, including the Material and Social Deprivation Index (MSDI) and Canadian Index of Multiple Deprivation (CIMD). For the MSDI, Gamache, Hamel, and Blaser proposed a Material Deprivation index based on level of income, education, and employment ratio, and a Social Deprivation index including proportion of people who are living alone, amongst other variables.^18,19^ Similarly, data from the Canadian Index of Multiple Deprivation (CIMD), a four-component classification based on the flux of neighborhood residents (Residential Instability), population of immigrants (Ethno-cultural composition), economic factors (economic dependency), and other housing and education factors (situational vulnerability) was also used.^16,17^ Finally, a neighborhood before-tax income was also derived. The deprivation index datasets were separated into quintiles, increments of 20%, with Q1 being the least deprived, and Q5 being the most deprived. Patients with known fixed addresses in the province were matched using postal codes associated with their place of residence to the Canadian deprivation index. The Supplemental Methods (Supplemental 1) describe the underlying data behind the CIMD and MSDI indices, as well as the methods used to match patient residence postal codes to deprivation indices used in this manuscript.

### Statistical analysis

Baseline patient characteristics and geographic variables were described using descriptive statistics. These characteristics were compared across patient community sizes. Patients with missing data were excluded from analyses for which their data was missing. Loss to follow up was not adjusted for as only 4 patients were lost to follow-up. Study size was determined by dataset size without *a priori* sample size calculations. The Supplemental Methods (Supplemental 1) further describes community size coding and reference variable selection.

For adjusted mRS analyses, 3 separate logistic regression models were used with the outcome variable of mRS dichotomized between 0–2 inclusive (a good outcome) compared to 3–6 inclusive (a poor outcome), and one of the following sets of covariates: the MSDI deprivation indices (material deprivation, social deprivation); the CIMD deprivation indices (Residential instability, ethno-cultural composition, economic dependency, situational vulnerability); and neighborhood before-tax income. Each analysis was adjusted for age, sex, distance to hospital, external hospital transfer status, home community size, presenting NIHSS, and last seen normal to arterial access time.

The primary analysis encoded each of the deprivation indices as a categorical variable (4 levels), with a sensitivity analysis encoding each as a continuous variable and using non-quintile data from CIMD and neighborhood before-tax income. For an unadjusted mRS analysis, multiple univariate logistic regressions were used with the same outcome variable but using each component of the MSDI deprivation indices, CIMD deprivation indices, and neighborhood before-tax income separately. The results of encoding these indices both as categorical and continuous variables are presented.

The same covariates used for mRS analysis (except that LSN to hospital time was used instead of LSN to arterial access, and NIHSS was treated as the outcome variable) were used to create multivariate linear regressions with the outcome variable of presenting NIHSS. Similarly, the same covariates used for mRS analysis were used to create multivariate ordinal regressions with the outcome variable of Thrombolysis in Cerebral Infarction (TICI) score. Statistical significance was set at p < 0.05. Statistical analysis was performed in R Version 4.4.3 (Vienna). Figure creation, mapping, and data processing were done using Python 3.11.3.

## Results

Of the 838 included patients, 793 (94.6%), had non-missing postal codes and met the inclusion criteria of having postal codes within Manitoba, Canada and completed mRS outcomes. (Of these, 834/838 (99.5%) had available mRS data). 773/793 (97.4%) had available CIMD data, 724/793 (91.2%) had available MSDI data, and 793/793 (100%) had neighborhood income data. The majority of patients (549/793 (69.2%)) were from communities of population size >100,000 (urban communities), with 171/793 (21.6%) of patients living in rural communities. Table 1 summarises the patient demographics. Age, baseline ASPECTS, distance to HSC, patient transfer status, and LSN to arterial access times differed with respect to regions with differing population sizes; notably, LSN to arterial access times appeared to be lowest in the Urban (i.e., Winnipeg) group (median = 3.72 h, IQR 2.75–6.83 h).

**Table 1.** Patient characteristics of the patient population by community size.

	Rural	Small population centre (1000–29,999)	Medium population centre (30,000–99,999)	Urban population centre (100,000+)	p
Number of Presenting Patients - n	171	53	20	549	
Age (years) - median (IQR)	71 [60–79.5]	72 [65–80]	72.5 [65.5–79.25]	75 [64–83]	**0** **.** **0198**
Sex (female) - n (%)	95/171 (55.56%)	27/53 (50.94%)	6/20 (30%)	277/548 (50.55%)	0.1726
NIHSS at baseline - median (IQR)	14 [10–18]	13 [8.75–18]	13.5 [9.25–16.5]	13 [8–18]	0.2252
Baseline ASPECTS score - median (IQR)	7 [6–9]	8 [6–8]	7 [6–8]	8 [7–9]	**0**.**0079**
Occlusion Location - n (%)					0.7975
M1	102/171 (59.65%)	35/53 (66.04%)	12/20 (60%)	306/548 (55.84%)	
M2	32/171 (18.71%)	6/53 (11.32%)	3/20 (15%)	130/548 (23.72%)	
ICA	21/171 (12.28%)	7/53 (13.21%)	3/20 (15%)	64/548 (11.68%)	
Basilar	15/171 (8.77%)	4/53 (7.55%)	2/20 (10%)	45/548 (8.21%)	
Other	1/171 (0.58%)	1/53 (1.89%)	0/20 (0%)	3/548 (0.55%)	
Neighborhood before-tax income - median (IQR)	54677 [48685–62813]	55420 [52359–64947]	57279 [51689–59824]	59020 [48360–69742]	0.1111
Distance to HSC in km - median (IQR)	81.83 [52.36–155.57]	54.17 [35.49–96.86]	200.39 [200.02–201.87]	6.58 [4.04–9]	**<0**.**0001**
Transfer status - n (%)					**<0**.**0001**
EMS	72/171 (42.11%)	19/53 (35.85%)	1/20 (5%)	404/548 (73.72%)	
External hospital	92/171 (53.8%)	30/53 (56.6%)	18/20 (90%)	124/548 (22.63%)	
Inpatient stroke	4/171 (2.34%)	2/53 (3.77%)	1/20 (5%)	12/548 (2.19%)	
Other	3/171 (1.75%)	2/53 (3.77%)	0/20 (0%)	8/548 (1.46%)	
LSN to puncture time (hours) - median (IQR)	5.5 [3.51–8.67]	5.22 [3.74–8.56]	7.75 [5.82–11.18]	3.72 [2.75–6.83]	**<0**.**0001**
EVT not aborted - n (%)	147/171 (85.96%)	45/53 (84.91%)	17/20 (85%)	458/548 (83.58%)	0.8987
TICI score - n (%)					0.605
Grade 0	19/169 (11.24%)	6/51 (11.76%)	3/18 (16.67%)	55/529 (10.4%)	
Grade 1	4/169 (2.37%)	0/51 (0%)	0/18 (0%)	16/529 (3.02%)	
Grade 2a	15/169 (8.88%)	8/51 (15.69%)	0/18 (0%)	42/529 (7.94%)	
Grade 2b	44/169 (26.04%)	9/51 (17.65%)	4/18 (22.22%)	139/529 (26.28%)	
Grade 2c	10/169 (5.92%)	1/51 (1.96%)	0/18 (0%)	21/529 (3.97%)	
Grade 3	77/169 (45.56%)	27/51 (52.94%)	11/18 (61.11%)	256/529 (48.39%)	

**Figure 1.**
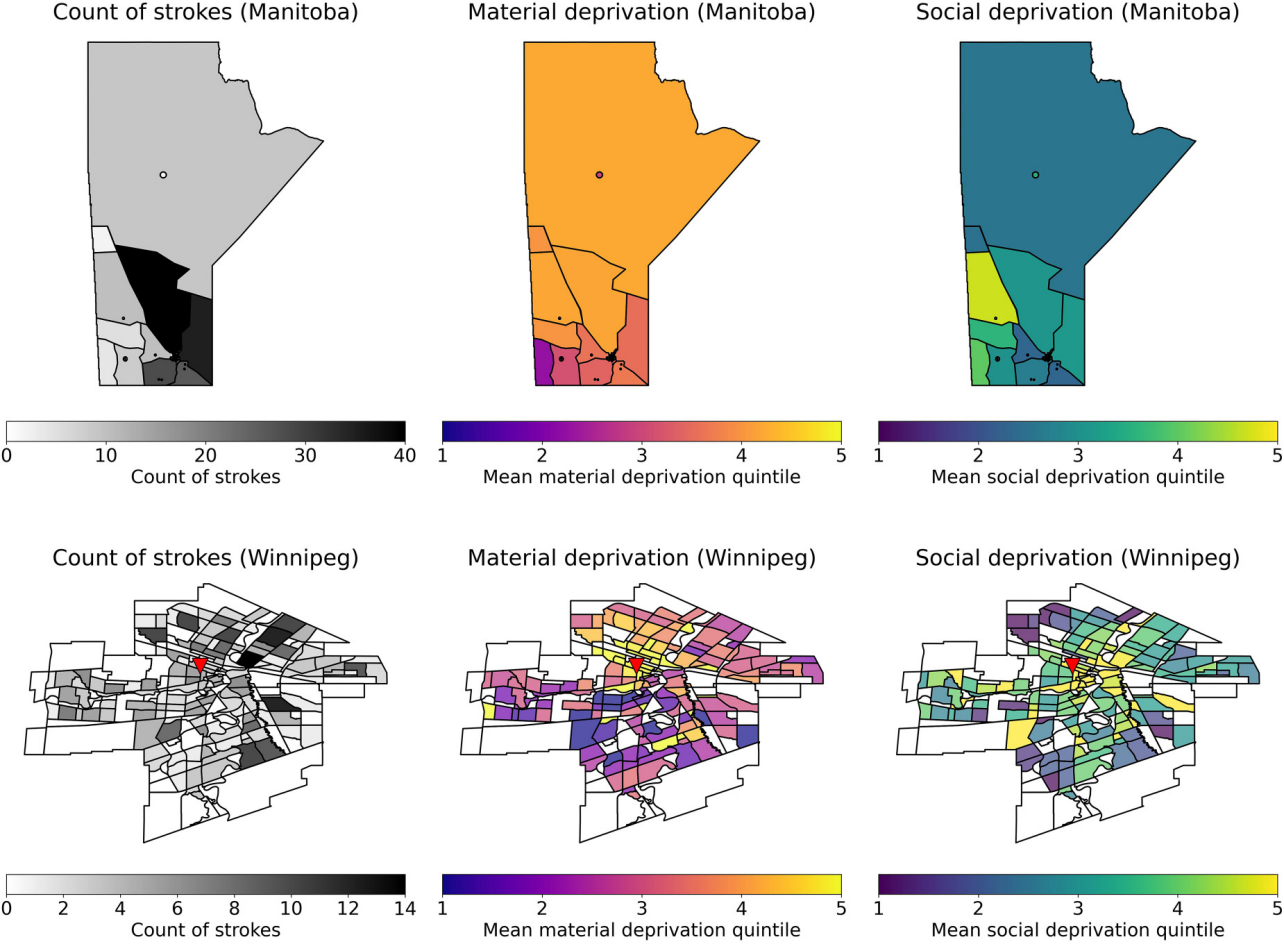
Visualization of MSDI deprivation indices within Manitoba, Canada and winnipeg, Manitoba. TOP: left - Heatmap of stroke count by postal code region in Manitoba; middle - mean material deprivation quintile; right - mean social deprivation quintile. BOTTOM: Left - Heatmap of stroke count by neighborhood in Winnipeg, Manitoba. Middle - Mean material deprivation quintile amongst stroke patients in each neighborhood. Right - Mean social deprivation quintile amongst stroke patients in each neighborhood. The triangle represents the location of Health Sciences Centre, Winnipeg.

Note that social deprivation quintiles, NIHSS, and outcome measures are separately visualized in Figure 1.

For MSDI deprivation indices, the median material deprivation quintile was 3 (IQR 2–4) and the median social deprivation quintile was 4 (IQR 2–5). (Note that these represent regional quintiles and reflect greater material deprivation in this population compared to the Prairies average). For CIMD deprivation indices, the median residential instability quintile was 4 (IQR 2–5), the median ethno-cultural composition quintile was 4 (IQR 3–5), the median economic dependency quintile was 4 (IQR 2–4), and the median situational vulnerability quintile was 3 (IQR 2–4).

**Figure 2.**
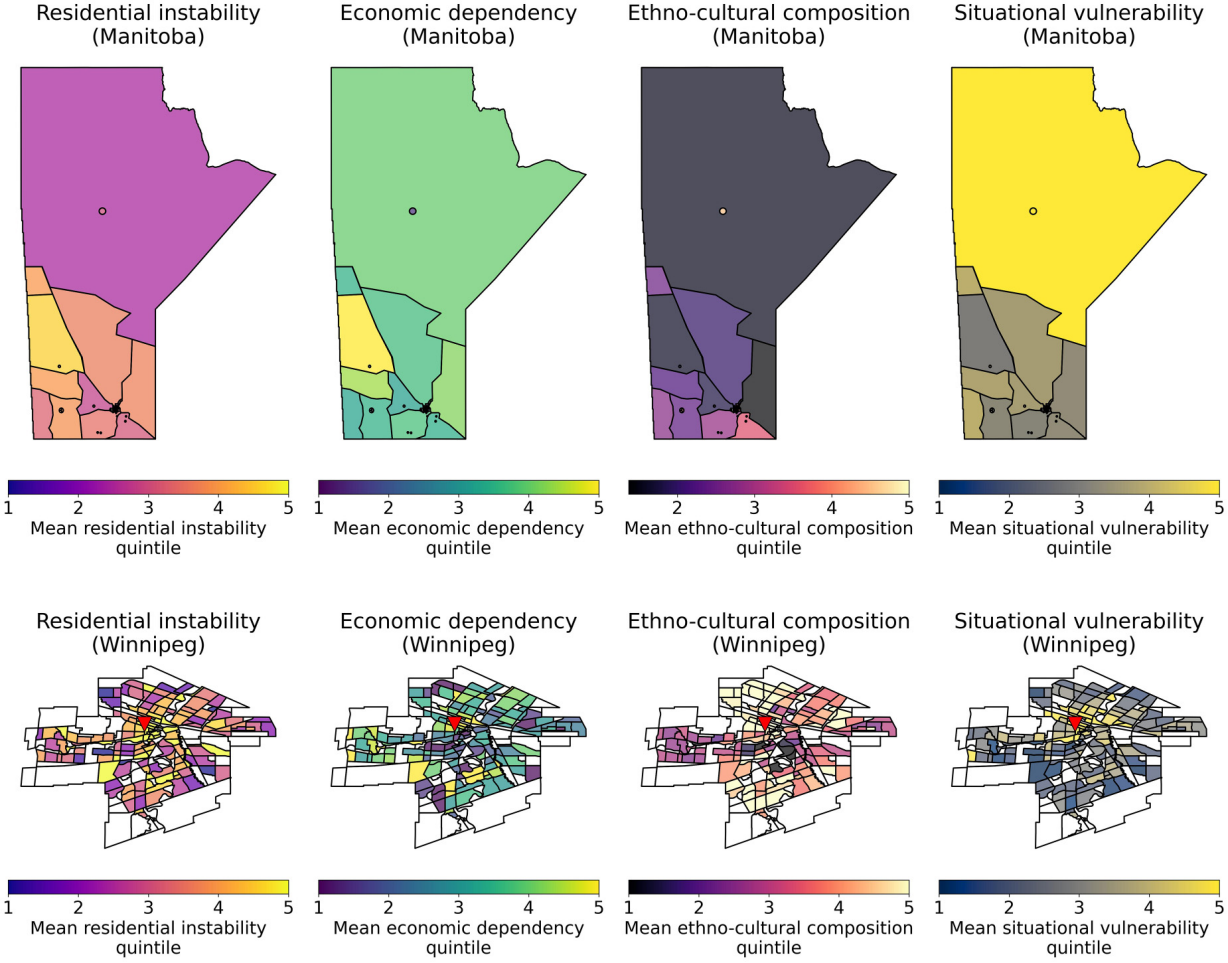
Visualization of CIMD deprivation indices within Manitoba, Canada and winnipeg based on the prairie specific index. The top row represents the mean deprivation index amongst stroke patients within postal code areas, while the bottom row represents the mean deprivation index amongst stroke patients within neighborhoods in Winnipeg. The triangle represents the location of Health Sciences Centre, Winnipeg.

Figures 1 and 2 visualize the geographic distribution of material and social deprivation within Winnipeg and within Manitoba and show a high prevalence of high-quintile MSDI material deprivation, MSDI economic dependency, and CIMD situational vulnerability in northern Manitoba and the downtown Winnipeg as compared to southern Manitoba and the periphery of Winnipeg. Similarly, the highest quintile MSDI social deprivation scores were located within downtown Winnipeg. Highest quintile ethno-cultural composition scores were found within Winnipeg in areas far from downtown Winnipeg and were lowest in rural areas. There were more patients in the Quintiles 4 and 5 (more deprived) categories compared to the Quintiles 1 and 2 (less deprived) categories in all studied CIMD and MSDI deprivation indices. Supplemental 2 graphically summarizes the statistical distribution of the MSDI index in the studied population, while Supplemental 3 graphically summarizes the distribution of the CIMD index in the studied population.

**Figure 3.**
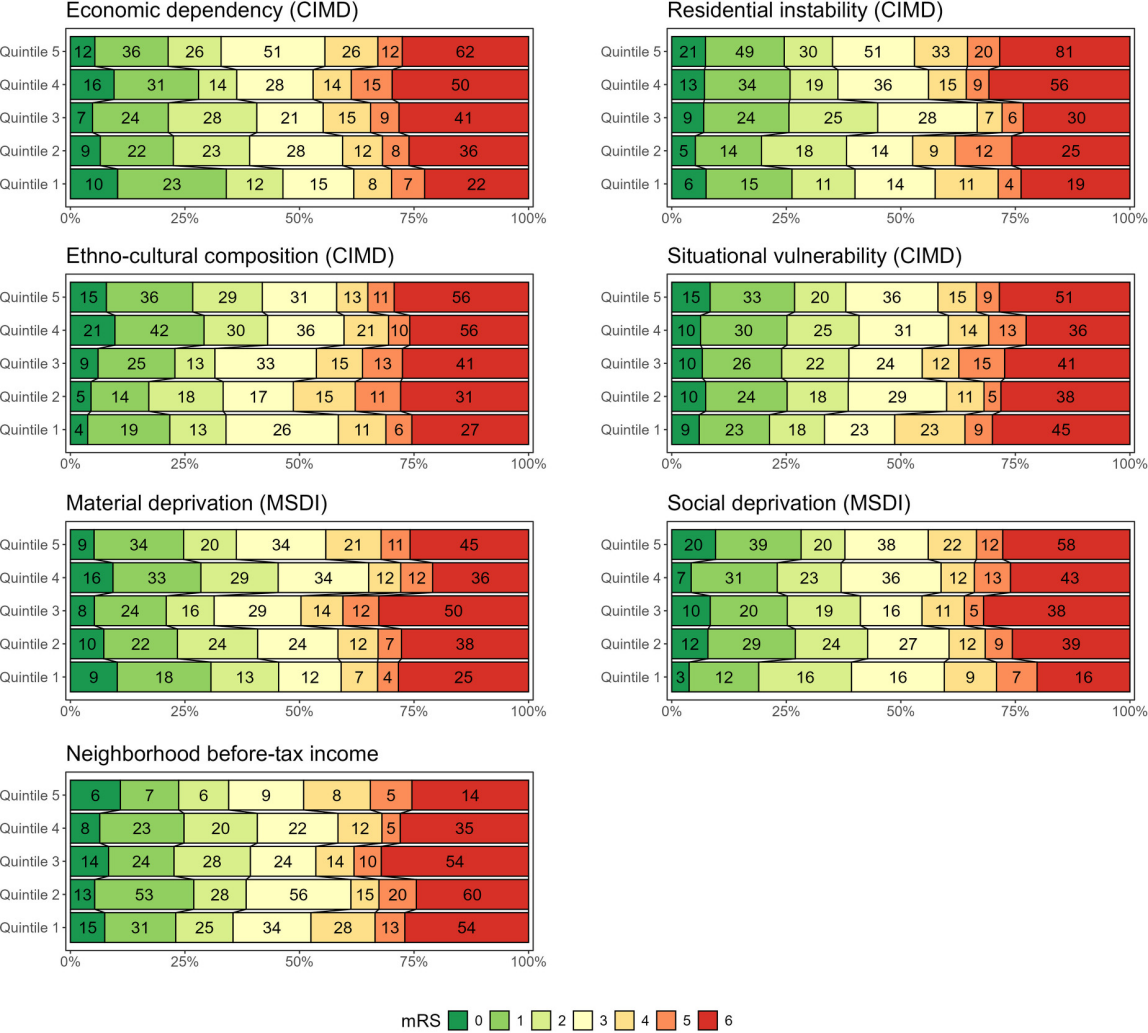
Distribution of primary outcome 90-day mRS scores examined across different CIMD, MSDI, and national before-tax income quintiles. Note that 773 patients had available CIMD data, 724 had MSDI data, and all had estimated neighborhood income data.

In adjusted logistic regression analyses, only the third quintile of economic deprivation in MSDI (OR 2.05 [95% CI 1.06, 4.02]) was significantly associated with poor mRS 3–6 outcomes at 90 days (Table 2). In unadjusted analyses, increased CIMD economic dependency scores (OR 1.18 [95% CI 1.03, 1.37]) and decreased CIMD ethno-cultural composition scores (OR 0.84 [95% CI 0.73, 0.98]) were associated with a higher odds of poor outcomes (Table 3). Grotta bars are also used to visually summarize unadjusted analyses (Figure 3). Older age, higher presenting NIHSS, and increased LSN to arterial access times were associated with worsened clinical outcomes in adjusted analyses (Table 2 - top section).

**Table 2.** Odds ratios for adjusted logistic regression analysis for the association between 90-day mRS (binarized to 0–2 and 3–6) and deprivation indices.

	OR [95% CI] MSDI Model	p	OR [95% CI] Income Model	p	OR [95%CI] CIMD Model	p
**Age (years)**	**1.05 [1.04, 1.07]**	**<0**.**0001**	**1.06 [1.04, 1.07]**	**<0**.**0001**	**1.06 [1.04, 1.07]**	**<0**.**0001**
Male sex	1.02 [0.7, 1.49]	0.9	1 [0.7, 1.43]	1	0.97 [0.67, 1.41]	0.89
**Presenting NIHSS**	**1.09 [1.06, 1.12]**	**<0**.**0001**	**1.09 [1.06, 1.13]**	**<0**.**0001**	**1.09 [1.06, 1.12]**	**<0**.**0001**
Distance to HSC (km)	1 [1, 1]	0.32	1 [1, 1]	0.33	1 [1, 1]	0.29
Non-HSC hospital transfer	0.96 [0.62, 1.49]	0.86	1.02 [0.67, 1.56]	0.91	1.06 [0.68, 1.65]	0.8
Patient community population (rural reference)						
1000–29,999	1.14 [0.51, 2.6]	0.75	1.15 [0.53, 2.53]	0.72	1.43 [0.62, 3.39]	0.41
30,000–99,999	1.65 [0.44, 7.05]	0.47	1.96 [0.61, 7.21]	0.28	1.97 [0.57, 7.69]	0.3
100,000+	0.96 [0.54, 1.71]	0.9	0.94 [0.55, 1.61]	0.82	1.2 [0.63, 2.29]	0.58
**LSN to arterial access time (hrs)**	**1.08 [1.03, 1.13]**	**0**.**00061**	**1.08 [1.03, 1.13]**	**0**.**00046**	**1.08 [1.03, 1.13]**	**0**.**00076**
Economic deprivation (Q1 reference)	Economic deprivation		Reverse-coded neighborhood before-tax income		Economic dependency	
Quintile 2	1.56 [0.79, 3.07]	0.2	0.64 [0.28, 1.43]	0.28	0.96 [0.49, 1.89]	0.9
Quintile 3	**2.05 [1.06, 4.02]**	**0**.**034**	0.83 [0.38, 1.79]	0.64	1.31 [0.67, 2.59]	0.43
Quintile 4	1.07 [0.55, 2.07]	0.85	0.8 [0.37, 1.68]	0.55	1.14 [0.59, 2.21]	0.7
Quintile 5	1.52 [0.78, 2.95]	0.22	0.94 [0.43, 2.02]	0.87	1.22 [0.62, 2.41]	0.56
Social deprivation (Q1 reference)	Social deprivation				Residential instability	
Quintile 2	0.83 [0.42, 1.63]	0.6			1.32 [0.6, 2.9]	0.49
Quintile 3	1.04 [0.51, 2.11]	0.92			0.87 [0.41, 1.83]	0.71
Quintile 4	1.34 [0.68, 2.65]	0.4			1.74 [0.85, 3.58]	0.13
Quintile 5	0.95 [0.48, 1.83]	0.87			1.4 [0.68, 2.88]	0.36
Ethno-cultural composition (Q1 reference)						
Quintile 2					1.31 [0.65, 2.65]	0.45
Quintile 3					1.27 [0.62, 2.6]	0.52
Quintile 4					0.71 [0.36, 1.37]	0.31
Quintile 5					0.76 [0.37, 1.56]	0.46
Situational vulnerability (Q1 reference)						
Quintile 2					1.2 [0.63, 2.26]	0.58
Quintile 3					1.45 [0.75, 2.8]	0.27
Quintile 4					1.13 [0.6, 2.14]	0.71
Quintile 5					1.24 [0.65, 2.4]	0.51
Model (N, p)	571	**<0**.**0001**	617	**<0**.**0001**	602	**<0**.**0001**

5 represents the least privileged quintile, while 1 represents the most privileged quintile. Bolded entries represent statistically significant covariates at p < 0.05.

**Table 3.** Odds ratios for unadjusted logistic regression analysis for the association between 90-day mRS (binarized to 0–2 and 3–6) and deprivation indices.

	OR [95% CI] Quintile 2	p	OR [95% CI] Quintile 3	p	OR [95% CI] Quintile 4	p	OR [95% CI] Quintile 5	p	OR [95% CI] Continuous	p
MSDI - Material deprivation	1.21 [0.7, 2.07]	0.5	1.82 [1.06, 3.14]	0.03	1 [0.6, 1.68]	0.99	1.47 [0.87, 2.47]	0.15	1.04 [0.93, 1.17]	0.44
MSDI - Social deprivation	0.86 [0.49, 1.5]	0.61	0.92 [0.51, 1.65]	0.79	1.1 [0.63, 1.91]	0.73	1.06 [0.62, 1.8]	0.82	1.05 [0.94, 1.17]	0.39
Neighborhood before-tax income	0.88 [0.6, 1.3]	0.53	0.85 [0.56, 1.3]	0.45	0.8 [0.5, 1.27]	0.34	1.04 [0.56, 1.98]	0.9	1 [1, 1]	0.69
CIMD - Economic dependency	1.35 [0.8, 2.28]	0.27	1.26 [0.75, 2.12]	0.38	1.52 [0.91, 2.53]	0.11	**1.77 [1.08, 2.87]**	**0**.**022**	**1.18 [1.03, 1.37]**	**0**.**023**
CIMD - Residential instability	1.08 [0.59, 1.98]	0.8	0.82 [0.46, 1.43]	0.48	1.17 [0.68, 2.01]	0.57	1.23 [0.74, 2.05]	0.42	1.13 [0.99, 1.28]	0.069
CIMD - Ethno-cultural composition	1.03 [0.58, 1.81]	0.92	1.12 [0.65, 1.89]	0.68	0.68 [0.42, 1.1]	0.12	0.71 [0.43, 1.16]	0.18	**0.84 [0.73, 0.98]**	**0**.**024**
CIMD - Situational vulnerability	0.8 [0.49, 1.3]	0.36	0.79 [0.49, 1.27]	0.34	0.72 [0.45, 1.15]	0.17	0.82 [0.52, 1.28]	0.38	0.96 [0.82, 1.13]	0.64

Bolded entries represent statistically significant covariates at p < 0.05. Quintile analyses were conducted using Prairie region deprivation index quintiles and a reverse-coded national neighborhood before-tax income quintile, each coded as categorical variables with reference category of quintile 1 (least deprived). Continuous analyses were separately conducted using Prairie region deprivation index quintiles (MSDI), neighborhood before-tax income, and Prairie region deprivation index Z-scores (CIMD) coded as continuous variables.

Factors associated with presenting NIHSS were analyzed in a linear regression analysis presented in Supplemental 4. Increased CIMD ethno-cultural composition quintiles were associated with decreased presenting NIHSS (Quintile 3: −2.22 [95% CI −4.29, −0.16], Quintile 4: −3.08 [95% CI −5.01, −1.15]) compared to Quintile 1. (This implies that decreased ethno-cultural composition is associated with higher presenting NIHSS.) Additionally, shorter times to hospital arrival (in hours) were associated with higher presenting NIHSS (−0.22/hr [95% CI −0.34, −0.09]). Supplemental 5 shows the results of an ordinal regression analysis with the outcome of TICI score post-angiography. There were no factors found to be associated with TICI score in this analysis, including socioeconomic status, geographic location, patient, or process factors.

## Interpretation

### Summary

In this provincial study of patients that underwent EVT within Manitoba (population 1.4 million, covering a 650,000 km^2^ study catchment area for EVT),^20^ indices of socioeconomic status were associated with poor outcomes on unadjusted analyses, but these findings were did not hold after adjusting for clinical and geographic variables. There was no clear relationship between 90-day mRS and patients’ community SES, or whether they presented from metropolitan or rural areas.

### Explanation of findings

Regarding the relationship between increased CIMD economic dependency scores and poor outcomes, one possibility is confounding by age [which is significantly associated with EVT outcomes in both this data and the literature, and also significantly positively associated with increased CIMD economic dependency (r = 0.11, p = 0.003)].^21,22^ Regarding the relationship between decreased ethno-cultural composition quintiles and poor outcomes, this may reflect the fact that decreased ethno-cultural composition quintiles were additionally associated with increased presenting NIHSS. Those communities with increased ethno-cultural composition quintiles were found primarily in Winnipeg, where several major hospitals including the primary site of the study is located. Furthermore, one association between deprivation indices and mRS was found, indicating a potential association between Quintile 3 MSDI material deprivation and mRS scores (OR = 2.05 [1.06, 4.02]). However, given that these findings were not replicable in the other quintiles or the other deprivation analyses, we did not consider this effect to be consistent.

Furthermore, there were no geographic, demographic, or socioeconomic factors predictive of angiographic outcomes as measured by TICI score, which did not support a difference in level of reperfusion achieved amongst patients from different geographic areas or socioeconomic status. Our findings do not suggest a consistent influence in post EVT outcomes based on socioeconomic status of the patients after adjusting for clinical, demographic and geographic factors in Manitoba. While these findings should be interpreted cautiously, one interpretation may indicate that potential inequities in stroke care in Manitoba occurred primarily prior to EVT. For example, the relationship between SES and outcomes post-EVT may have been confounded on unadjusted analysis by the observed relationship between higher age and higher economic dependency scores and lower ethno-cultural composition scores and higher NIHSS scores. Furthermore, the relationship between SES and between angiography initiation time was not examined in this study but is hypothesized to also represent a key confounding variable.

Our findings are consistent with prior findings regarding SES in stroke care, but demonstrate that these findings generalise to patients who have received EVT. Lower SES is consistently associated with increased morbidity and mortality post AIS.^23^ The association between SES and presenting severity has also been previously described; for example, Ghoneem et al. also found that lower SES was associated with higher presenting NIHSS, and also resulted in increased long-term disability post-stroke and estimated that the association between SES and NIHSS accounted for 64% of the association between SES and long-term disability.^23,24^ While SES is measured differently throughout the literature, composite Canadian deprivation indices have been studied in AIS. For instance, MSDI had previously been investigated as a factor in access to EVT.^14^ In this context, this study presents new findings with regards to the association between CIMD ethno-cultural composition and outcomes, a finding which had not been demonstrated within Canada.

### Limitations

One significant limitation was that the selection of patients for thrombectomy compared to EVT (which was investigated in the literature) could not be evaluated due to the nature of our data. The AIS literature suggests that SES-based disparities in patient selection for EVT are significant, even within areas with universal healthcare.^25^ Furthermore, neighborhood socioeconomic factors likely correlate with but may not truly reflect the actual social or economic factors experienced by a given patient. Notably, patients with missing postal codes were excluded; however, this introduces a type of selection bias as patients with missing postal codes due to homelessness (i.e., likely high levels of deprivation) were not included in this study. Another limitation is the generalizability of these findings, as data is from a single Canadian province (Manitoba).

### Future directions

Several future research directions are currently underway to address the limitations of this study. Because these findings were only applicable to a single Canadian province, a study using retrospective socioeconomic data encompassing the entire Canadian cohort is currently being conducted. Another key area of research involves investigating factors associated with poor outcomes in Manitoba and their relationship with geography and SES. In particular, increased angiography initiation time was a key factor associated with poor outcomes on an adjusted basis; however, the correlation between SES and angiography initiation time was not investigated in this study. Further research will also explore factors associated with time to angiography initiation in this population.

## Conclusion

Ischemic stroke patients undergoing EVT in Manitoba between 2015 and 2024 showed an unadjusted association between increased CIMD economic dependency scores and poor outcomes. Adjusted analyses showed no consistent associations between neighborhood socioeconomic or geographic factors and clinical or procedural outcomes. While further work is needed to elucidate the mechanism behind these findings, one potential explanation may be that the observed disparities in outcomes post-EVT were at least partially attributable to a relationship between SES and the adjustment variables of age, time to angiography initiation, and presenting severity.

## Supplemental Material

sj-zip-1-ine-10.1177_15910199251396174 - Supplemental material for Associations between Canadian deprivation indices and acute stroke outcomes post endovascular thrombectomy - A retrospective cohort study Supplemental material, sj-zip-1-ine-10.1177_15910199251396174 for Associations between Canadian deprivation indices and acute stroke outcomes post endovascular thrombectomy - A retrospective cohort study by Matthew C So, Nishita Singh, Johanna M Ospel, A Zohaib Siddiqi, Nada Dahroug, Santhosh Annayappa, Kamran Zahid, Susan Alcock, Roman Marin, Ankur Wadhwa, Claudia Candale-Radu, Anurag Trivedi, Esseddeeg Ghrooda, Naveed Akhtar, Mohammed Suheel, Joseph Silvaggio, Jai Shankar and Nima Kashani in Interventional Neuroradiology
