# Supplementary material for: Associations between Canadian deprivation indices and acute stroke outcomes post endovascular thrombectomy - A retrospective cohort study
Source: Interv Neuroradiol. 2025 Dec 2:15910199251396174. Online ahead of print. doi: 10.1177/15910199251396174 (PMC12672281; doi:10.1177/15910199251396174)
Supplement: sj-zip-1-ine-10.1177_15910199251396174 - Supplemental material for Associations between Canadian deprivation indices and acute stroke outcomes post endovascular thrombectomy - A retrospective cohort study [file sj-zip-1-ine-10.1177_15910199251396174.zip › Appendix4.docx]

|  | Coefficient [95%CI]  MSDI Model | p | Coefficient [95%CI]  Income Model | p | Coefficient [95%CI]  CIMD Model | p |
| --- | --- | --- | --- | --- | --- | --- |
| Age (years) | 0.02 [-0.02-0.06] | 0.39 | 0.02 [-0.02-0.06] | 0.24 | 0.03 [-0.01-0.07] | 0.21 |
| Male sex | -0.31 [-1.42-0.81] | 0.59 | -0.49 [-1.54-0.57] | 0.36 | -0.6 [-1.68-0.47] | 0.27 |
| Distance to HSC (km) | 0 [0-0.01] | 0.41 | 0 [0-0.01] | 0.37 | 0 [-0.01-0.01] | 0.64 |
| Non-HSC hospital transfer | 0.24 [-1.12-1.6] | 0.73 | 0.13 [-1.15-1.4] | 0.84 | 0.28 [-1.02-1.59] | 0.67 |
| Patient community population |  |  |  |  |  |  |
| 1,000-29,999 | -0.16 [-2.53-2.22] | 0.9 | -0.35 [-2.6-1.9] | 0.76 | 0.67 [-1.75-3.09] | 0.59 |
| 30,000-99,999 | -1.12 [-5.2-2.95] | 0.59 | -1.31 [-4.73-2.1] | 0.45 | -0.01 [-3.63-3.6] | 0.99 |
| 100,000+ | -1.09 [-2.83-0.65] | 0.22 | -1.41 [-3.01-0.19] | 0.084 | -0.25 [-2.14-1.63] | 0.79 |
| **LSN to hospital arrival time (hrs)** | **-0.19 [-0.32- -0.06]** | **0.0032** | **-0.22 [-0.34- -0.09]** | **0.00051** | **-0.22 [-0.34- -0.09]** | **0.00057** |
| Economic deprivation (Q1 reference) * | Economic deprivation | | Reverse-coded neighborhood before-tax income | | Economic dependency | |
| Quintile 2 | -0.87 [-2.91-1.17] | 0.4 | -0.23 [-2.66-2.2] | 0.86 | 0.04 [-1.95-2.02] | 0.97 |
| Quintile 3 | -0.11 [-2.08-1.86] | 0.91 | 0.34 [-1.99-2.68] | 0.77 | -0.47 [-2.43-1.5] | 0.64 |
| Quintile 4 | 0.25 [-1.72-2.21] | 0.8 | -0.11 [-2.37-2.16] | 0.93 | -0.02 [-1.94-1.9] | 0.99 |
| Quintile 5 | 0.6 [-1.38-2.57] | 0.55 | 0.06 [-2.24-2.36] | 0.96 | 0.79 [-1.16-2.75] | 0.43 |
| Social deprivation (Q1 reference) ** | Social deprivation | |  |  | Residential instability | |
| Quintile 2 | 0.91 [-1.18-3] | 0.39 |  |  | -1.06 [-3.39-1.27] | 0.37 |
| Quintile 3 | -0.66 [-2.8-1.49] | 0.55 |  |  | -1.84 [-4.05-0.37] | 0.1 |
| Quintile 4 | 0.45 [-1.61-2.52] | 0.67 |  |  | -1.84 [-3.94-0.26] | 0.085 |
| Quintile 5 | 0.06 [-1.97-2.1] | 0.95 |  |  | -2.1 [-4.22-0.02] | 0.052 |
| Ethno-cultural composition (Q1 reference) |  |  |  |  |  |  |
| Quintile 2 |  |  |  |  | -1.47 [-3.48-0.54] | 0.15 |
| Quintile 3 |  |  |  |  | **-2.22 [-4.29- -0.16]** | **0.035** |
| Quintile 4 |  |  |  |  | **-3.08 [-5.01- -1.15]** | **0.0018** |
| Quintile 5 |  |  |  |  | -1.84 [-3.91-0.23] | 0.081 |
| Situational vulnerability (Q1 reference) |  |  |  |  |  |  |
| Quintile 2 |  |  |  |  | 0.24 [-1.61-2.1] | 0.8 |
| Quintile 3 |  |  |  |  | 0.77 [-1.12-2.66] | 0.42 |
| Quintile 4 |  |  |  |  | 0.98 [-0.93-2.89] | 0.31 |
| Quintile 5 |  |  |  |  | 1.04 [-0.88-2.96] | 0.29 |
| Model | 570 | 0.17 | 619 | 0.054 | 605 | 0.059 |

**Supplemental Data 4:** Linear regression coefficients for the association between presenting NIHSS and deprivation indices. 5 represents the least privileged quintile, while 1 represents the most privileged quintile. Bolded entries represent statistically significant covariates at p < 0.05.
