# Supplementary material for: Associations between Canadian deprivation indices and acute stroke outcomes post endovascular thrombectomy - A retrospective cohort study
Source: Interv Neuroradiol. 2025 Dec 2:15910199251396174. Online ahead of print. doi: 10.1177/15910199251396174 (PMC12672281; doi:10.1177/15910199251396174)
Supplement: sj-zip-1-ine-10.1177_15910199251396174 - Supplemental material for Associations between Canadian deprivation indices and acute stroke outcomes post endovascular thrombectomy - A retrospective cohort study [file sj-zip-1-ine-10.1177_15910199251396174.zip › Appendix5.docx]

|  | OR [95% CI]  MSDI Model | p | OR [95% CI]  Income Model | p | OR [95% CI]  CIMD Model | p |
| --- | --- | --- | --- | --- | --- | --- |
| Age (years) | -0.01 [-0.02, 0] | 0.13 | -0.01 [-0.02, 0] | 0.1 | -0.01 [-0.02, 0] | 0.18 |
| Male sex | -0.11 [-0.43, 0.2] | 0.48 | -0.15 [-0.45, 0.16] | 0.35 | -0.18 [-0.49, 0.14] | 0.26 |
| Presenting NIHSS | 0 [-0.03, 0.02] | 0.77 | 0 [-0.03, 0.02] | 0.71 | 0 [-0.03, 0.02] | 0.87 |
| Distance to HSC (km) | 0 [0, 0.01] | 0.066 | 0 [0, 0] | 0.23 | 0 [0, 0.01] | 0.11 |
| Non-HSC hospital transfer | -0.01 [-0.38, 0.37] | 0.96 | 0.08 [-0.28, 0.44] | 0.66 | 0.12 [-0.26, 0.49] | 0.55 |
| Patient community population (rural reference) |  |  |  |  |  |  |
| 1,000-29,999 | 0.41 [-0.3, 1.14] | 0.27 | 0.4 [-0.28, 1.11] | 0.25 | 0.42 [-0.32, 1.18] | 0.28 |
| 30,000-99,999 | 0.19 [-1.1, 1.62] | 0.78 | 0.56 [-0.58, 1.81] | 0.35 | 0.22 [-0.98, 1.51] | 0.73 |
| 100,000+ | 0.47 [-0.03, 0.98] | 0.067 | 0.38 [-0.08, 0.85] | 0.11 | 0.44 [-0.12, 1] | 0.12 |
| LSN to arterial access time (hrs) | 0 [-0.04, 0.03] | 0.88 | -0.01 [-0.04, 0.03] | 0.74 | -0.01 [-0.05, 0.02] | 0.52 |
| Economic deprivation (Q1 reference) * | Economic deprivation | | Reverse-coded neighborhood before-tax income | | Economic dependency | |
| Quintile 2 | 0.41 [-0.18, 0.99] | 0.17 | 0.28 [-0.41, 0.96] | 0.42 | -0.24 [-0.83, 0.34] | 0.41 |
| Quintile 3 | 0.01 [-0.55, 0.57] | 0.97 | 0.11 [-0.55, 0.76] | 0.74 | -0.38 [-0.96, 0.2] | 0.2 |
| Quintile 4 | 0.43 [-0.15, 1] | 0.14 | 0.08 [-0.56, 0.7] | 0.81 | -0.49 [-1.07, 0.08] | 0.091 |
| Quintile 5 | 0 [-0.57, 0.56] | 1 | 0.26 [-0.4, 0.9] | 0.44 | -0.29 [-0.88, 0.29] | 0.33 |
| Social deprivation (Q1 reference) ** | Social deprivation | |  |  | Residential instability | |
| Quintile 2 | -0.34 [-0.94, 0.24] | 0.25 |  |  | -0.27 [-0.96, 0.4] | 0.43 |
| Quintile 3 | -0.42 [-1.04, 0.19] | 0.18 |  |  | -0.15 [-0.82, 0.51] | 0.65 |
| Quintile 4 | -0.45 [-1.06, 0.14] | 0.14 |  |  | -0.48 [-1.12, 0.15] | 0.14 |
| Quintile 5 | -0.19 [-0.79, 0.39] | 0.52 |  |  | -0.55 [-1.19, 0.09] | 0.094 |
| Ethno-cultural composition (Q1 reference) |  |  |  |  |  |  |
| Quintile 2 |  |  |  |  | -0.21 [-0.78, 0.36] | 0.47 |
| Quintile 3 |  |  |  |  | -0.15 [-0.75, 0.46] | 0.64 |
| Quintile 4 |  |  |  |  | 0.19 [-0.37, 0.75] | 0.51 |
| Quintile 5 |  |  |  |  | -0.07 [-0.68, 0.53] | 0.81 |
| Situational vulnerability (Q1 reference) |  |  |  |  |  |  |
| Quintile 2 |  |  |  |  | 0.09 [-0.44, 0.63] | 0.73 |
| Quintile 3 |  |  |  |  | -0.33 [-0.87, 0.2] | 0.22 |
| Quintile 4 |  |  |  |  | 0.05 [-0.5, 0.6] | 0.87 |
| Quintile 5 |  |  |  |  | 0.19 [-0.38, 0.76] | 0.51 |
| Model | 560 | 0.304 | 602 | 0.703 | 587 | 0.442 |

**Supplemental Data 5**: Ordinal regression coefficients for the association of TICI score with social, geographic, clinical, and demographic covariates across the entire dataset. 5 represents the least privileged quintile, while 1 represents the most privileged quintile. Bolded entries represent statistically significant covariates at p < 0.05.
