# Supplementary material for: Associations between Canadian deprivation indices and acute stroke outcomes post endovascular thrombectomy - A retrospective cohort study
Source: Interv Neuroradiol. 2025 Dec 2:15910199251396174. Online ahead of print. doi: 10.1177/15910199251396174 (PMC12672281; doi:10.1177/15910199251396174)
Supplement: sj-zip-1-ine-10.1177_15910199251396174 - Supplemental material for Associations between Canadian deprivation indices and acute stroke outcomes post endovascular thrombectomy - A retrospective cohort study [file sj-zip-1-ine-10.1177_15910199251396174.zip › Appendix1.docx]

**Supplmental Data 1: Supplemental Methods**

Gamache, Hamel, and Blaser proposed a series of certain key indicators to create the Material and Social Deprivation Index for each dissemination area, including: The proportion of the population aged 15 years and over without a high school diploma or equivalent; the employment to population ratio for the population 15 years and over; the average income of the population aged 15 years and over; the proportion of the population aged 15 and over living alone; the proportion of the population aged 15 and over who are separated, divorced or widowed; the proportion of single-parent families, which were standardised according to the age and sex structure of the Canadian population. These indicators were combined using principal component analysis and two main components were identified, which were thought to correspond to a material and social component. The material component was shown to reflect low income, education, and employment ratio, while the social component reflected a larger proportion of people who are separated, divorced, widowed, living alone, or in a single parent family. The MSDI dataset as provided was separated into quintiles with Q1 being the least deprived, and Q5 being the most deprived compared to other neighborhoods in the Prairies region. An edited version of the 2021 tables provided by Gamache, Hamel, and Blaser was used for postal code matching.

Similarly, data from Statistics Canada was used to create the Canadian Index of Multiple Deprivation (CIMD). The residential instability index was devised as a measure of the movement of neighborhood inhabitants, including the proportion of apartment buildings, proportion of persons living alone. Economic dependency was devised as a marker of the workforce and dependence on sources of income other than employment. Ethno-cultural composition was associated with the community makeup of immigrant populations, while situational vulnerability included the proportion of Indigenous population, proportion of homes needing major repairs, population without a high-school diploma, single-parent families, and median value of homes. The CIMD dataset as provided was separated into similar quintiles as above, and dissemination indices were combined using data from the PCCF 8B+ to obtain CIMD scores at the postal code level. The outputs included Prairie-level quintiles, as well as raw Z-scores for each neighborhood. Additionally, neighborhood before-tax income was derived for each dissemination area. A reverse-coded national quintile (i.e. a 5th quintile income is a 1st quintile deprivation, which is least deprived) was used for quintile analysis, and the raw neighborhood before-tax income was used for continuous analysis.

Patients with valid postal codes associated with their place of residence were matched to a deprivation index as described above, using the following algorithm. Firstly, dissemination indices were matched to postal codes for which there was a single corresponding dissemination area. This left postal codes which were associated with multiple dissemination areas. For some postal codes, the proportion of the population living in each dissemination area was known, and the weighted mean of each deprivation index (by population) was determined. Finally, for some postal codes, the proportion of the population living in each dissemination area was not known, and a uniform mean of each deprivation index was assigned to the postal code. The resulting values were rounded to the nearest integer to obtain the quintile deprivation index (compared to Prairie regional data) for each postal code, for all MSDI and CIMD indices as well as neighborhood income metrics. If this algorithm failed for a full 6-digit postal code, a match using a larger neighborhood using the first 5 digits were attempted. If the first 5 characters could not find a match, the first four digits were attempted. This continued until 2 digit postal code regions were attempted.

Patient community sizes included the following groups: rural (<1000), small population centre (1,000-29,999), medium population centre (30,000-99,999), and urban population centre (100,000+). Data was descriptively compared using medians and interquartile ranges for continuous variables, and proportions for categorical variables. Categorical variables were compared between quintiles using the χ2 test, while continuous variables were compared using the Student’s t-test. For each analysis, the 1st quintile of deprivation (the least deprived) was chosen as the reference variable and the “medium or large city” (i.e. Winnipeg, Manitoba for all cases) was chosen as the reference variable for the size of population.
