# Supplementary figures and images for: Associations between Canadian deprivation indices and acute stroke outcomes post endovascular thrombectomy - A retrospective cohort study
Source: Interv Neuroradiol. 2025 Dec 2:15910199251396174. Online ahead of print. doi: 10.1177/15910199251396174 (PMC12672281; doi:10.1177/15910199251396174)

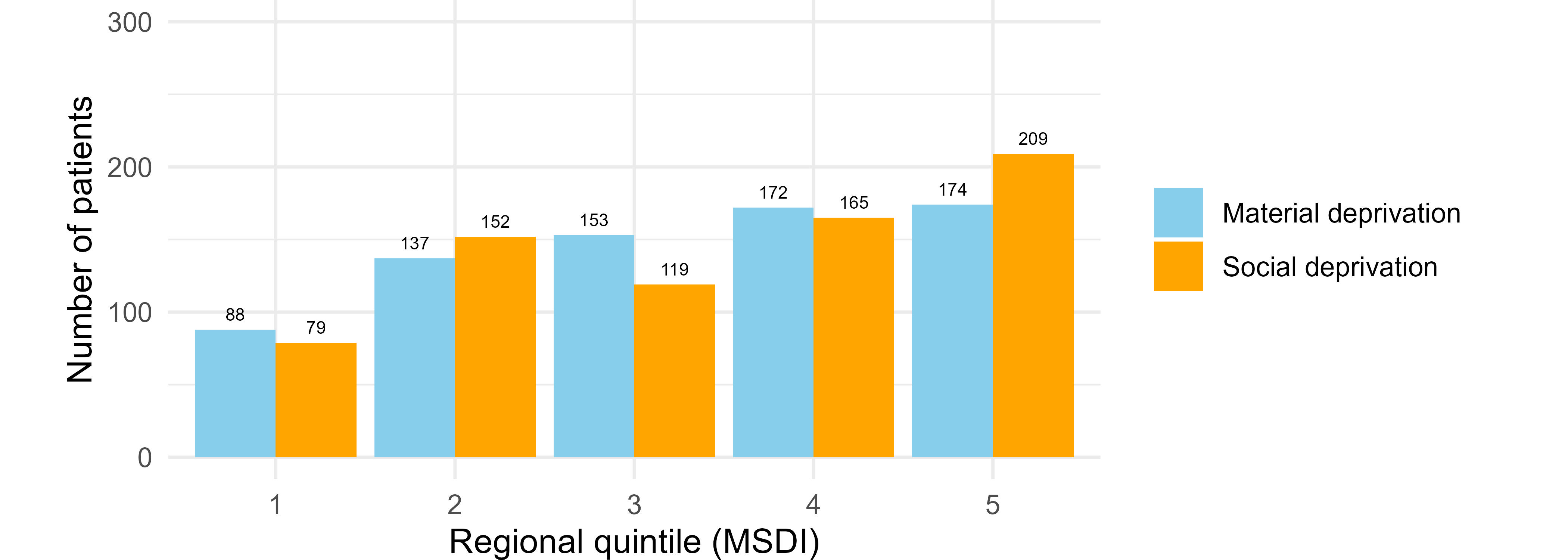

Supplement: sj-zip-1-ine-10.1177_15910199251396174 - Supplemental material for Associations between Canadian deprivation indices and acute stroke outcomes post endovascular thrombectomy - A retrospective cohort study [file sj-zip-1-ine-10.1177_15910199251396174.zip › appendix2.jpg]

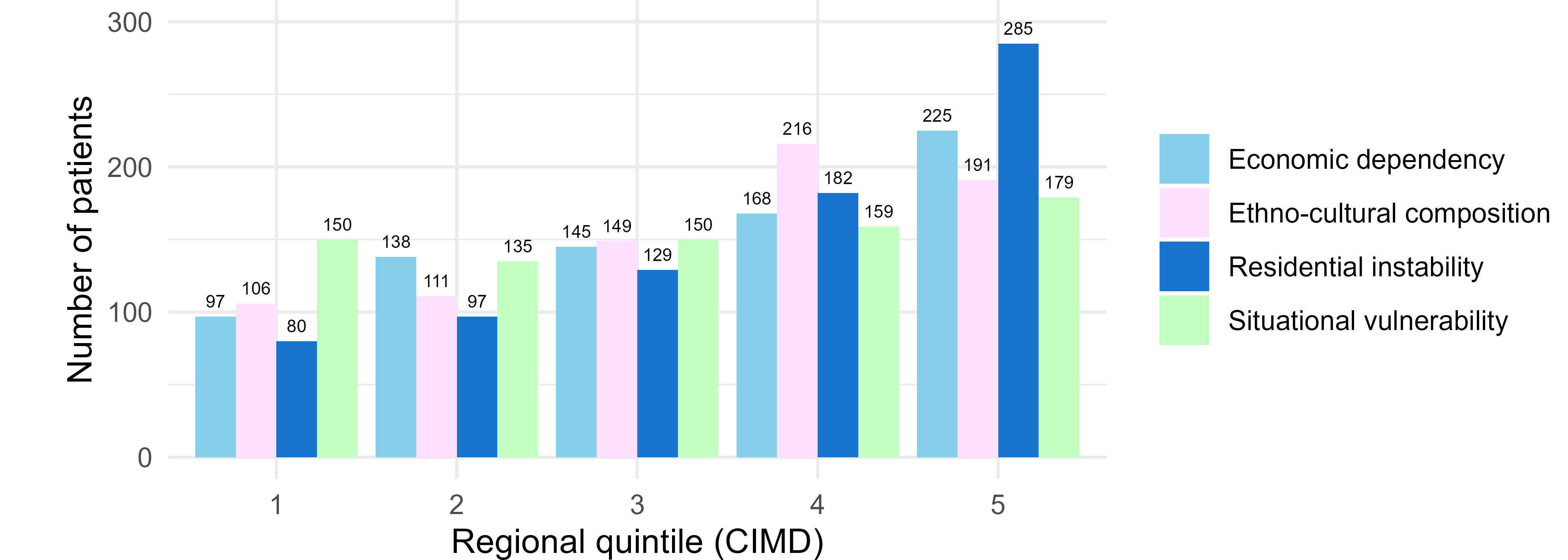

Supplement: sj-zip-1-ine-10.1177_15910199251396174 - Supplemental material for Associations between Canadian deprivation indices and acute stroke outcomes post endovascular thrombectomy - A retrospective cohort study [file sj-zip-1-ine-10.1177_15910199251396174.zip › appendix3.jpg]
